# Comparison of two ActiGraph accelerometer generations in the assessment of physical activity in free living conditions

**DOI:** 10.1186/1756-0500-5-187

**Published:** 2012-04-25

**Authors:** Jérémy Vanhelst, Jacques Mikulovic, Gilles Bui-Xuan, Olivier Dieu, Thomas Blondeau, Paul Fardy, Laurent Béghin

**Affiliations:** 1 Centre d’Investigation Clinique, CIC-PT-9301-Inserm-CH&U, Lille 59037, France; 2 ER3S, EA4110, Université Lille Nord de France, Dunkerque, France; 3 ER3S, Université d'Artois, Villeneuve d'Ascq, France; 4 Department of Family Nutrition, and Exercise Sciences (FNES), Queens College, Flushing, New York, USA; 5 Unité Inserm U995, Université Lille Nord de France, Lille, France

**Keywords:** Accelerometry, Physical activity, Assessment, Equivalence

## Abstract

**Background:**

The aim of this study was to compare physical activity measured using GT1M ActiGraph and GT3X ActiGraph accelerometers in free living conditions.

**Findings:**

Twenty-five adults wore GT1M and GT3X Actigraph accelerometers simultaneously during a typical weekday of activity. Data were uploaded from the monitor to a computer at the end of test (one day). Previously established thresholds were used for defining time spent at each level of physical activity, physical activity was assessed at varying intensities comparing data from the two accelerometers by ANOVA and Bland and Altman statistical analysis. The concordance correlation coefficient between accelerometers at each intensity level was 0.99. There were no significant differences between accelerometers at any of the activity levels. Differences between data obtained in minutes with the GT1M accelerometer and the GT3X monitor were to 0.56, 0.36, 0.52 and 0.44% for sedentary, light, moderate and vigorous, respectively. The Bland and Altman method showed good agreement between data obtained for the two accelerometers.

**Conclusions:**

Findings suggest that the two accelerometers provided similar results and therefore the GT3X may be used in clinical and epidemiological studies without additional calibration or validation studies.

## Findings

### Introduction

Health benefits of physical activity (PA) have been demonstrated for many chronic diseases. For example moderate to vigorous intensity activity (MVPA) has been shown to decrease obesity and lower total cholesterol and blood pressure [1].

Accurate measurement of PA is essential in developing intervention strategies. Physical activity questionnaires (PAQ), diaries, observations*,* indirect calorimetry, double-labeled water (DLW), heart rate monitors and accelerometry have been used [2-4]. Because of the limitation of PAQ methods and the high cost and subject burden associated with direct observation and doubly labeled water, accelerometry has become the method of choice for objective, valid and reliable measurement in adults [5].

The uniaxial ActiGraph accelerometer (ActiGraph^TM^, Pensacola, CA) is widely accepted as valid in assessing PA in laboratory and FLC [6-8], and has been used in epidemiological studies [9,10]. Even if the triaxial accelerometer measures physical activity during walking with more precision than the uniaxial accelerometer [11], a recent study showed that there is no difference between uniaxial and triaxial accelerometers in the measurement of PA [12]. However, recently, the manufacturer improved the GT1M for a triaxial accelerometer (GT3X). This device may also be used in uniaxial mode (GT1M mode). It is important to determine if there are discrepancies between the two models in assessing time spent in different intensities of PA, using previous thresholds established with old versions of accelerometers, or if the development of new physical activity threshold values is necessary. If the GT3X accelerometer, in uniaxial mode, has different results than the old generation, then studies that use the GT3X cannot be compared with data from previous studies. Additional studies will be necessary to calibrate and validate the new device.

To date, there are no published studies comparing the new generation ActiGraph accelerometer (GT3X) and its predecessor (GT1M). The purpose of our study is to compare the time spent at different intensities of PA by simultaneous measurements involving the ActiGraph GT1M and the GT3X accelerometers.

## Methods

Twenty-five healthy sport science students were recruited. Physical characteristics of the subjects are described in Table1. Subjects were required to pass a medical examination to exclude contraindications for participating in the study. The purpose and objectives were carefully explained to each subject and written informed consent was obtained. The study was approved by the local Ethics Committee (Comité de Protection des Personnes).

**Table 1:** **Physical characteristics of subjects (*n*** **= 25)**

	**Males**	**Females**
**N**	14	11
**Age (*yr*)**	25.3 ± 4.8	25.5 ± 4.4
**Weight (*Kg*)**	72.1 ± 10.1	61.0 ± 9.8
**Height (*cm*)**	177.2 ± 5.5	170.8 ± 6.6
**BMI (Kg/m**^**2**^**)**	22.9 ± 2.8	20.8 ± 2.1

Weight was measured to the nearest 0.1 kg using an electronic scale (Oregon Scientific®, GA 101, USA). Height was measured without shoes to the nearest 0.1 cm using a stadiometer (Seca®, Hamburg, Germany).

Accelerometers were calibrated according to manufacturer specification. The epoch interval used was set at one min and output was expressed as mean counts per minute. All participants wore the two ActiGraph accelerometers (GT1M & GT3X in uniaxial mode) simultaneously, at the level of the back with the same elastic belt and adjustable buckle, during a typical week day. Subjects were instructed to remove the devices during swimming, showering, and bathing. The accelerometers recorded activity during the day, and were removed at night. Data were uploaded from the monitor to a computer after the period test. The following PA thresholds were used: sedentary activity, 0 to 99 counts·min^–1^, light activity 100–1951 counts·min^–1^, moderate activity 1952–5723 counts·min^–1^, and vigorous activity ≥ 5724 counts·min^–1^[13]. The same accelerometers were used for all participants.

All analyses were performed using SAS software version 9.2 (SAS Institute Inc., Cary, NC 25513). Physical activity was measured and analyzed in counts per minute. ANOVA compared PA between the two accelerometers. P values <0.05 were considered statistically significant.

Quantitative variables were described by mean and 95% confidence intervals. Reproducibility between GT1M and GT3X accelerometers was assessed with intraclass correlation coefficient (ICC) at each intensity. The scale used for interpretation of concordance was previously described [14].

The Bland and Altman method was used to test agreement of data output between GT1M and GT3X [15]. The analysis measures bias as estimated from mean differences, the 95% confidence interval for bias, and the limits of agreement, ± 2 standard deviations of the difference. The GT1M was used as the reference for analysis because it had been validated previously and calibrated to assess the PA intensity and/or estimate the energy expenditure during normal daily conditions [6,7,13,16].

### Results and discussion

Participants wore accelerometers an average of 903 ± 137 min. Mean PA during the recording time was 584 ± 205 counts·min^–1^ for the GT1M® and 595 ± 206 counts·min^–1^ for the GT3X®. No significant differences in PA were found between genders (p = 0.22).

The concordance correlation coefficient between accelerometers at each intensity was 0.99 (Table2). There were no significant differences in intensity between accelerometers at the four intensities (Table2). Differences between accelerometers never exceeded 0.56%.

**Table 2:** Time spent in different intensity of PA expressed in minutes per day for the both accelerometer generations (n = 25)

**Intensity**	**Mean [95% IC]**		**Mean difference [95% IC]**	**ICC**
	**GT1M**	**GT3X**		
**Sedentary**	683.40 [625.97; 740.83]	682.84 [415.17; 950.51]	0.56 [−0.84; 1.96] ^†^	0.99
**Light**	132.92 [80.82; 185.02]	132.56 [80.62; 184.52]	0.36 [−1.14; 1.86] ^††^	0.99
**Moderate**	47.28 [28.75; 65.81]	47.80 [29.06; 66.54]	- 0.52 [−1.38; 0.34] *	0.99
**Vigorous**	39.44 [23.98 ; 54.90]	39.88 [24.25; 55.51]	- 0.44 [−0.96; 1.84] **	0.99

* P = 0.25, ** P = 0.11, ^†^ P = 0.44, ^††^ P = 0.64.

**Figure 1:**
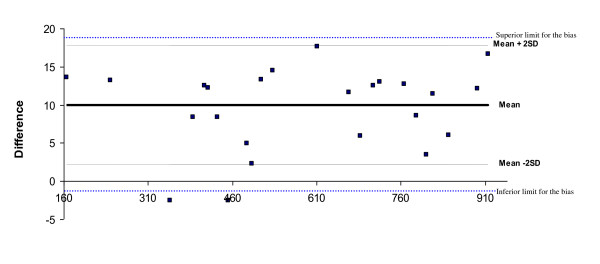
Difference of total mean counts assessed between GT1M and GT3X accelerometers.

Agreement was assessed at different intensities. Mean differences were within the limits of agreement and most data points were within the limits of agreement of bias (Figure1).

Agreement between the devices was also compared for their ability to identify participants who met the MVPA activity guideline of 60 min per day. Participants whose PA intensities met PA guidelines were identical for the two accelerometers.

Selecting the ActiGraph accelerometer model is an important issue for researchers. The present study showed a concordance and no significance difference between data output obtained by the new vs. the previous generation.

Studies have compared different ActiGraph accelerometers in laboratory or FLC [8,17-19]. Using a motorized table with a wide range of amplitude and frequencies to assess three generations of ActiGraph monitors, significant differences in activity counts between generations of accelerometers were reported [8]. Results of the study showed inter-accelerometer variability consistently better with GT1M compared with the 7164 or 71256 accelerometers for all frequencies, with a mean difference of ± 20%. Therefore, conclusions about differences among three generations in the present study have to be considered with caution because of intermonitor variability [8]. A study comparing adolescents using the GT1M ActiGraph (Version 1) and Model 7164 in FLC found no significant difference in time spent in moderate and vigorous physical activity when using the same epoch length, although differences were observed in sedentary and light-intensity activity [17]. Compared with Model GT1M, Model 7164 exhibited significantly less time as sedentary and more time as light-intensity activity (P < 0.001). Corder et al (2007) concluded that data from the GT1M can be compared with historical data using average counts per minute, and the two models are comparable when measuring time spent in MVPA in children using the same epoch length [17]. Differences in time spent at different intensities of PA were not significant. Kozey et al (2010) compared the ActiGraph accelerometer model 7164 with the ActiGraph GT1M during self-paced locomotion at three speeds of walking [19] and concluded that the GT1M is comparable to Model 7164 when estimating habitual activity intensities [19]. A study comparing activity counts between the ActiGraph 7164 and the three versions of the GT1M at given walking and running speeds concluded that there were no statistically significant differences between outputs from the accelerometers, suggesting that researchers can select any of the four ActiGraph accelerometers for measuring PA [18].

The present study adds information, comparing the last version of GT1M with the last version of ActiGraph (GT3X), and confirms results previously published with other generations of ActiGraph accelerometers. Findings suggest that the two devices assess PA similarly and that data from the two devices are comparable in studies of PA patterns. The two devices were equivalent in identifying subjects meeting the 60 min of MVPA · day^–1^.

A high correlation was reported between the GT1M ActiGraph accelerometer and oxygen consumption [6,7]. Results from the present study suggest that the GT3X accelerometer is a valid instrument for measuring PA. Further studies are suggested for assessing the capacity of the device to measure PA, especially intra and inter instrument reliability.

Although results of the study provide important information regarding the use of accelerometers, there are limitations to consider. One limitation relates to the number of accelerometers used in the study. Because of financial and practical constraints only one accelerometer of each model was used. Wearing multiple accelerometers simultaneously is possible but would be difficult for the subject and could influence PA in free living conditions. Perhaps a complementary study using mechanical set-up, e.g., a motion table where several accelerometers are assessed together, would be helpful in controlling for confounding effects of monitor placement and type of activity. Results from the present study, however, show good reliability and a concordance value of 0.99 at each intensity, sedentary, light, moderate and vigorous. A possible second limitation is the time period used to monitor activity. Perhaps the difference between the two accelerometers would be greater if data were collected for a longer period of time. Finally, the thresholds of Freedson et al, were chosen for the present study because of frequency of use found in the literature. We cannot exclude the possibility that PA would have been different had we used other thresholds.

In summary, our findings suggest that the GT3X accelerometer in mode GT1M may be used in clinical and epidemiological investigations without additional calibration or validation studies. Moreover, studies using the new generation of accelerometer can be compared to those using the GT1M.

## Competing interests

The authors declare that they have no competing interests.

## Authors’ contributions

JV, PF, OD and TB carried out study and drafted the manuscript. JM, GBX and LB participated in the design of the study, and contributed with critical review of the manuscript. All authors read and approved the final manuscript.
